# Systemic anticancer therapy during end of life in head and neck squamous cell carcinoma patients. A retrospective single center study.

**DOI:** 10.1007/s00432-025-06297-5

**Published:** 2025-08-29

**Authors:** Simone Rota, Silvia Buriolla, Andrea Franza, Stefano Cavalieri, Cristiana Bergamini, Salvatore Alfieri, Imperia Nuzzolese, Elena Colombo, Arianna Ottini, Benedetta Lombardi Stocchetti, Giacomo Massa, Augusto Caraceni, Lisa Licitra, Carlo Resteghini

**Affiliations:** 1 Department of Head and Neck Medical Oncology, Fondazione IRCCS - Istituto Nazionale dei Tumori, Milano, Italy; 2 Department of Oncology and Hemato-Oncology, University of Milan, Milano, Italy; 3 Department of Clinical Sciences and Community Health, Università degli Studi di Milano, Milan, Italy; 4 Palliative Care, Pain Therapy and Rehabilitation Unit, Fondazione IRCCS Istituto Nazionale dei Tumori, Milan, Italy

**Keywords:** Head and neck squamous cell carcinoma, Systemic anticancer treatment, Palliative care, Treatment timing, Aggressive cancer subtypes, End of life

## Abstract

**Purpose:**

Systemic anticancer treatments (SACTs) are used in advanced cancer stages to control disease and improve survival. However, their use at the end of life (Eol) can lead to side effects. This study aims to assess the clinical implications of SACTs near the Eol in patients with recurrent/metastatic head and neck squamous cell carcinoma (R/M HNSCC).

**Methods:**

This is an observational retrospective study conducted at Fondazione IRCCS Istituto Nazionale dei Tumori di Milano. R/M HNSCC patients treated from 2016 to 2024 were included and classified based on the timing of their last SACT relative to death: within 30 days (Cohort 1), and more than 30 days before death (Cohort 2). Cohort 1 was further subdivided into treatments given 15–30 days (1a) or within 14 days (1b) before death. We assessed performance status, survival outcomes, time from last therapy administration to death, fatal acute adverse events, and palliative care referral rates.

**Results:**

One hundred fifty-five patients were evaluable for the analysis. Patients receiving last anticancer therapy within 30 days of death exhibited worse overall survival compared to those who received their last treatment earlier (HR = 0.69; 95% CI 0.48–1.00; *p* = 0.047). Fatal acute events occurred in 31% of cases, with respiratory failure and major bleeding as leading causes.

**Conclusion:**

SACTs within 30 days of death correlates with worse prognosis and more aggressive disease. Major bleeding may be treatment-related when administered within 14 days of death. These findings emphasize the need for careful patient selection and early palliative care integration.

**Supplementary Information:**

The online version contains supplementary material available at 10.1007/s00432-025-06297-5.

## Introduction

Systemic anticancer treatments comprise different types of agents, including chemotherapy, targeted therapies and immunotherapy. In advanced stages of disease these interventions lead to disease control with potential benefit on survival and symptomatic relief. However, when administered at the end of life, systemic anticancer treatments expose patients to harmful side effects with limited benefit (Canavan et al. 2022), increase in intensive care unit admissions and in-hospital mortality rates. (Geyer 2023; McPherson et al. 2018; Stanley 1980; Schnipper et al. 2012; Crawford et al. 2021). This led to the development of field-specific guidelines by the American and European Societies of Clinical Oncology, advocating for the cessation of systemic anticancer treatments in cases of ECOG PS ≥ 3, and in scenarios where no discernible benefit emerges across the first three lines of systemic therapies. (Schnipper et al. 2012; Crawford et al. 2021). Nonetheless, the fundamental principle of 'do no harm', as enshrined in the Hippocratic Oath, underscores these guidelines and remains a guiding tenet in making treatment decisions at the end of life.

Recent literature data showed that in a cohort of unselected solid cancer patients the rate of systemic anticancer treatments use in the last 30 days of life was 39%, with a slight substitution of chemotherapy with immune checkpoint inhibitors and target therapies since 2015 (Canavan et al. 2022; Parikh et al. 2019; Riaz et al. 2020; Johnson et al. 2020).

Up to now, few evidence is available for patients with recurrent / metastatic head and neck squamous cell carcinoma. Indeed, these patients have a short oncological history due to their frailty, as they are characterized by a persistent risk of fatal acute adverse events (e.g.; carotid blowout syndrome, major tumor bleeding, acute respiratory failure and sepsis (Bergamini et al. 2021; Ferreyro and Munshi 2019; Mirabile et al. 2015)), the highest suicide rates among solid cancers and a lack of effective late-line treatments.

Thus, identifying and understanding the risk of fatal acute events is crucial for reducing the rate of inappropriate systemic anticancer treatments use at the end of life.

Finally, no literature data are available regarding the clinical outcomes of recurrent / metastatic head and neck patients receiving systemic treatment within the last 30 days compared to those receiving it earlier, both in terms of overall survival (OS) and progression free survival (PFS).

The study aims to characterize the head and neck cancer population receiving systemic anticancer treatments at the end of life (defined as the last 30 days) compared to those receiving it earlier.

## Material and methods

### Patients population

This retrospective single-center study included recurrent / metastatic head and neck squamous cell carcinoma patients treated with systemic anticancer treatments for recurrent or metastatic disease from 2016 to 2024 in a tertiary cancer center in Northern Italy with dedicate head and neck cancer units (Fondazione IRCCS Istituto Nazionale dei Tumori di Milano).

Selection criteria were: diagnosis of recurrent or metastatic head and neck squamous cell carcinoma, ECOG PS ≤ 2 at baseline (before the start of first line of systemic therapy), absence of concomitant active neoplasms, available date of death and last systemic anticancer treatments administration and receipt of immunotherapy in at least one line of treatment during the patient journey. ECOG PS was recorded at two distinct time points: at baseline (prior to the initiation of first-line systemic treatment) and immediately before the start of the last line of therapy. Baseline ECOG was employed in the descriptive statistics, univariate, and multivariate Cox regression analyses, whereas ECOG assessed prior to the last administration of systemic treatment was used in the logistic regression analysis.

The following clinical data were collected for each subject: gender, age at time of death, presence of a caregiver (prior to the initiation of first-line systemic treatment and before the start of last line of therapy), primary tumor site (oral cavity, oropharynx, larynx, hypopharynx or carcinoma of unknown primary), comorbidities according to Charlson’s index, type of systemic therapy administered in the period of interest (CT; IO; target therapy), occurrence of events with fatal outcomes (e.g. carotid blow-up syndrome, major tumor bleeding and respiratory failure due to pneumonia, aspiration, and pneumonitis), date of last dose of systemic anticancer treatment, referral to palliative service (home- or hospice-based), and date of death. Furthermore, values of neutrophils and lymphocytes referred to the five days before first line of systemic treatment initiation were collected.

Patients were divided into two cohorts based on the timing of their last systemic anticancer treatment administration relative to death:

1. Cohort 1: Patients who received last systemic anticancer treatment within the last 30 days of life;
2. Cohort 2: Patients who received last systemic anticancer treatment earlier.

Further subdivisions were made to refine the analysis:

1. Cohort 1a: Patients who received last systemic anticancer treatment between 15 and 30 days before death.
2. Cohort 1b: Patients who received last systemic anticancer treatment within the last 14 days of life.

Primary endpoint was to compare OS and PFS between cohorts 1 and 2. Secondary endpoints were the rate of systemic anticancer treatments use; the overall median time to death from the last dose of cancer therapy administered across the entire cohort; the rate of referral to palliative care services and the rate of fatal acute events.

The study was conducted in compliance with the principles outlined in the Declaration of Helsinki and received approval from the Institutional Review Board of the Fondazione IRCCS Istituto Nazionale dei Tumori di Milano, Italy (local reference number: INT 85/23).

### Statistical analyses

Overall survival (OS) was defined as the time from the first systemic therapy administration for recurrent / metastatic disease to death from any cause. Progression-Free Survival (PFS) was defined as the time from the start of the first, second, and third line of systemic therapy (PFS1, PFS2, and PFS3, respectively) to disease progression. To examine baseline differences between groups, Pearson’s Chi square test or Fisher’s exact test were used, as appropriate.

Survival analyses were performed using the Kaplan–Meier method and Cox proportional hazards regression. Univariable Cox models were used to assess the association between the timing of the last systemic anticancer treatment (≤ 30 vs > 30 days before death) and both overall survival (OS) and progression-free survival (PFS). Univariable Cox regression analysis was also used to assess the effect of different baseline factors on OS. Variables significantly associated with survival outcomes at the univariable analysis were then fitted in a multivariable Cox proportional hazards regression model to identify independent predictors of OS. Hazard ratios with the corresponding 95% confidence intervals were provided for Cox’s proportional hazards regression models. The proportional hazards assumption was assessed using Schoenfeld residuals (global test *p* = 0.43) and by visual inspection of log–log survival plots; no significant violations were observed.

In addition, a logistic regression analysis was performed to explore clinical predictors of receiving systemic anticancer treatment more than 30 days before death. The model included sex, ECOG performance status before the last line of systemic therapy and caregiver presence before the last line of systemic therapy as independent variables. All covariates were categorical. Odds ratios (ORs) with 95% confidence intervals were calculated.

All statistical tests were two-tailed, and a *p* < 0.05 was considered statistically significant. Statistical analyses were performed using R (version 4.4.1). Response rate was defined as the proportion of patients who achieved a complete or partial response per RECIST criteria, excluding stable disease.

## Results

### Patients’ characteristics

**Fig. 1:**
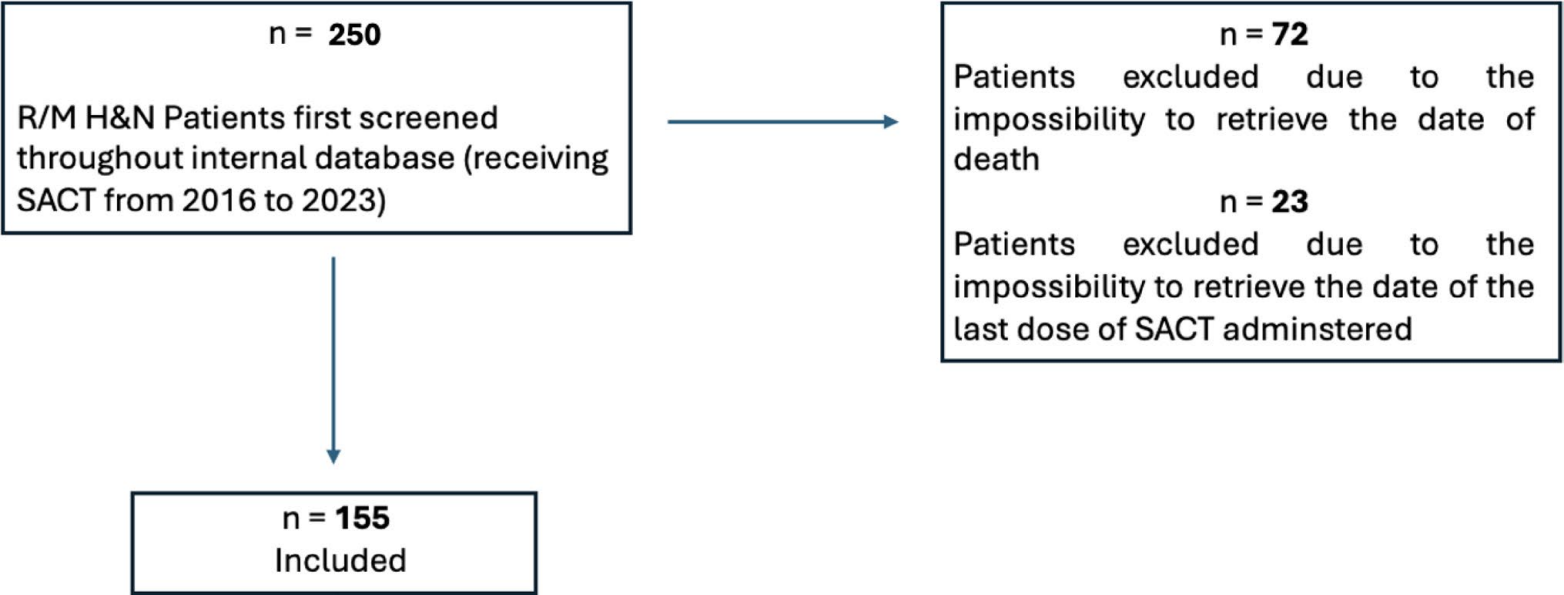
Consort of included patients

Overall, 155 patients affected by recurrent / metastatic head and neck squamous cell carcinoma cancer were included with the prevalence of male (73%). Ninety-five patients were excluded due to the lack of the date of death or the date of the last systemic anticancer treatment administration, mainly because treatment continuation occurred at external institutions and complete documentation was unavailable (Fig. 1).

Median age at the time of death was 55.8 years. Patients characteristics are shown in Table 1.

**Table 1:** Patients characteristics

Characteristics N = 155^1^			
Sex		Target therapy*	1 (1%)
Female	42 (27%)	NA	6 (4%)
Male	113 (73%)	Last systemic anticancer treatment administration timing	
Median age at time of death (years)	56 (27–89)	Before the last 30 days of life	116 (75%)
Site of primary tumor		Between the last 30 and 15 days of life	19 (12%)
Oral Cavity	74 (48%)	During the last 14 days of life	20 (13%)
Oropharynx	34 (22%)	Acute events	
HPV +	17 (50%)	Yes	48 (31%)
HPV	17 (50%)	No	37 (24%)
Larynx	28 (18%)	Not available (NA)	70 (45%)
Hypopharynx	18 (11%)	Type of acute event	
CUP	1 (1%)	Respiratory failure	17 (11%)
Charlson comorbidity index		Major bleeding	11 (7%)
0–5	1 (1%)	Sepsis	6 (4%)
6–10	138 (88%)	Hypercalcemia	4 (3%)
11–15	12 (8%)	Pneumonia	2 (1%)
NA	4 (3%)	SIADH	2 (1%)
Caregiver		Pneumothorax	1 (1%)
Yes	108 (70%)	Cardiac arrest	1 (1%)
No	29 (19%)	Hyponatriemia	1 (1%)
NA	18 (11%)	Stroke	1 (1%)
Type of last systemic anticancer treatment		Pulmonary thromboembolism	1 (1%)
IO	79 (50%)	Unknown	107 (68%)
Chemotherapy (CT)	68 (44%)	Median time to death from last cancer therapy (days)	49.0 (19.5–61.5)
CT + immunotherapy (IO)	1 (1%)		

^*^No antiangiogenic agents were employed

^1^n / N (%); Mean (SD)

The most common primary tumor site was the oral cavity (48%), followed by the oropharynx (22%), larynx (18%) and hypopharynx (11%); among oropharynx cases, 50.0% were HPV positive.

Median time to death from the last systemic anticancer treatment administration was 49 days (19.5–61.5).

One-hundred-twenty-one (78%) patients were referred to a residential palliative care service (home-care or hospice), while 15 (10%) had no contact with a palliative care specialist. In 19 (12%) cases data were not available. Moreover, in the last 30 and 14 days of life, the rate of admission in palliative home-care increased from 27% (47 cases) to 56% (87 cases), respectively. For hospice referrals, the rise was from 6% (9 cases) to 22% (34 cases). Data on patients followed as palliative care outpatients before the last systemic anticancer treatment are not available.

In the overall population, one third of patients experienced a fatal acute adverse event, with respiratory failure being the most common (11%), followed by major bleeding (7.1%) and sepsis (3.9%). Regarding intensity of care during end of life, hospitalization rates were similar between patients who received their last systemic anticancer treatment between 15–30 days and those who received it in the last 14 days of life (30% vs 39%, *p* = 0.73).

### Survival outcomes according to systemic anticancer treatment timing

**Fig. 2:**
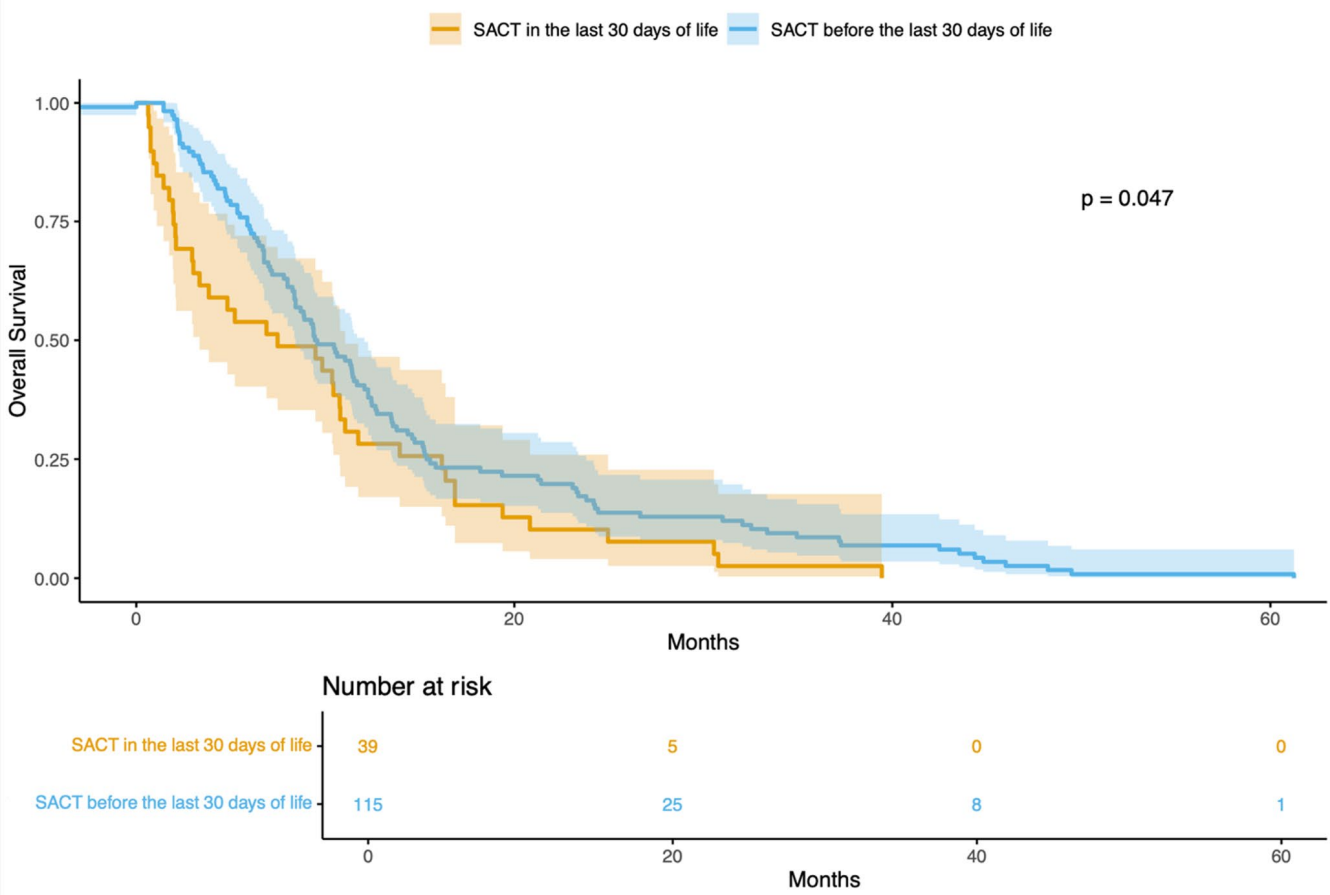
Kaplan–Meier curves for OS divided into cohort 1 and cohort 2 based on the timing of patients' last systemic anticancer treatment

Figure 2 illustrates OS in the entire population, divided into cohort 1 and cohort 2 based on the timing of patients' last systemic anticancer treatment administration.

Patients who received their last dose of systemic anticancer treatment more than 30 days prior to death demonstrated significantly superior OS compared to those who received treatment within the last 30 days of life (HR = 0.69, 95% CI 0.48–1.00; *p* = 0.047).

The effects of baseline ECOG PS (before the start of first line of systemic therapy), Charlson comorbidity index, gender, and the presence of a caregiver on overall survival were then investigated in both univariate and multivariate analyses. In the univariate analysis, ECOG showed a significant association with survival (HR = 1.67, 95% CI 1.16–2.40, *p* = 0.006), reflecting poorer survival with higher values. This association remained significant in the multivariate model (HR = 1.93, 95% CI 1.26–2.96, *p* = 0.002), confirming its role as a strong predictor of survival. Other variables considered did not reach statistical significance.

**Fig. 3:**
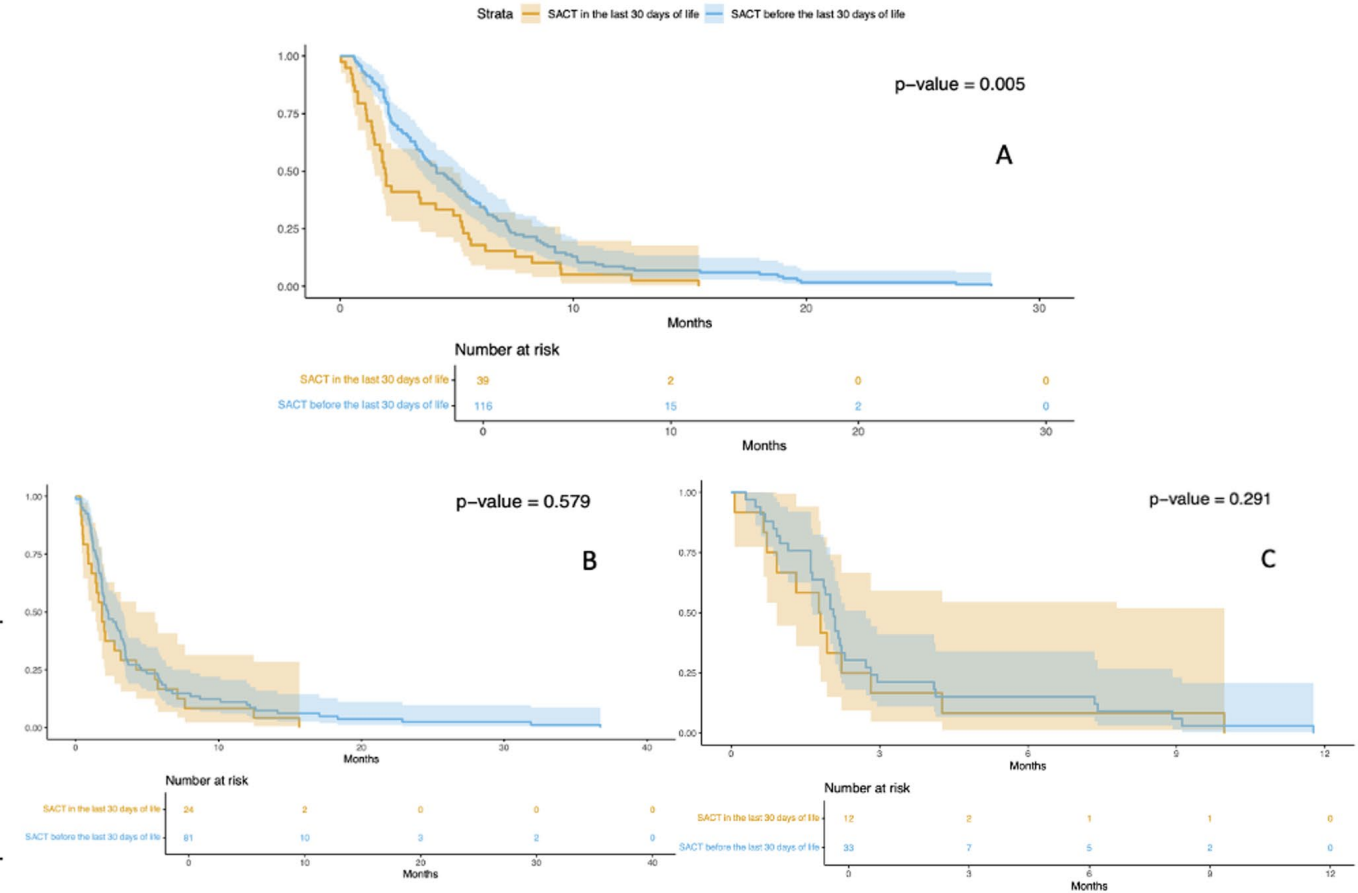
Kaplan–Meier curves for PFS divided into cohort 1 and cohort 2 based on the timing of patients' last systemic anticancer treatment. PANEL **A** First line progression free survival (PFS1). PANEL **B** Second line progression free survival (PFS2). PANEL C: third line progression free survival (PFS3)

Figure 3 illustrates the PFS in patients who received systemic anticancer treatment within the last 30 days of life compared to those who received systemic anticancer treatment more than 30 days prior to death. In Panel A (representing PFS1), a statistically significant difference is observed between the two groups (*p*-value 0.005). This suggests that, in the first-line setting, patients who received systemic anticancer treatment within the last 30 days of life had a shorter PFS compared to others. Response rates were 28% for the first group and 39% for the second one. In contrast, in Panels B and C, referring to PFS during the second and third lines of anticancer treatments, respectively, the differences between groups are not statistically significant.

Table 2 shows a comparison between the characteristics of patients who had undergone systemic therapy within the last 30 days of life and earlier, respectively.

**Table 2:** Patients characteristics in two different timing of end of life systemic anticancer treatment (30 days to death vs earlier)

Characteristics	Cohort 1, N = 39^1^	Cohort 2, N = 116^1^	*p*-value^2^
Gender			0.1
Female	15 (38%)	27 (23%)	
Male	24 (62%)	89 (77%)	
Baseline ECOG			0.2
1	28 (72%)	97 (84%)	
2	11(28%)	19 (16%)	
Charlson Comorbidity Index			0.1
0–5	1 (3%)	0 (0%)	
6–0	35 (90%)	103 (89%)	
11–15	1 (3%)	11 (9%)	
NA	2 (4%)	2 (2%)	
HPV status (oropharynx)			0.9
Yes	6 (15%)	15 (13%)	
No	7 (18%)	20 (17%)	
NA (not oropharynx)	26 (67%)	81(70%)	
Total number of systemic lines			0.5
1	12 (31%)	33 (28%)	
2	15 (38%)	48 (41%)	
3	12 (31%)	35 (30%)	
Acute adverse events			0.1
Yes	17 (44%)	31 (27%)	
No	9 (23%)	28 (24%)	
Data not available (NA)	13 (33%)	57 (49%)	
Type of acute event			0.6
Respiratory failure	6 (15%)	11 (9%)	
Major bleeding	5 (13%)	6 (5%)	
Sepsis	4 (10%)	10 (9%)	
Stroke	1 (3%)	1 (1%)	
Pulmonary thromboembolism	1 (3%)	0 (0%)	
NA	22 (56%)	88 (76%)	
Type of last therapy			0.3
CT	18 (46%)	52 (45%)	
ICI	18 (46%)	61 (53%)	
Target Therapy	1 (3%)	0 (0%)	
NA	2 (5%)	3 (2%)	
Presence of a caregiver			0.03
Yes	30 (77%)	78 (67%)	
No	9 (23%)	20 (17%)	
NA	0 (0%)	18 (16%)	

^1^n (%); Median (IQR)

^2^Fisher's exact test; Welch Two Sample t-test; Pearson's Chi-squared test

The distribution of patients by gender was not significantly different between those receiving systemic anticancer treatment in the last 30 days of life and those receiving it earlier (*p* = 0.1), as was the case for ECOG performance status (*p* = 0.2).

The occurrence fatal acute events was higher in patients receiving systemic anticancer treatment in the last 30 days (44%) compared to those receiving it earlier (31%), though this difference did not reach statistical significance (*p* = 0.1).

Moreover it was possible to obtain a baseline pre-first line treatment neutrophil and lymphocyte values for 60 out of 155 patients. The ratio showed a value of 7.5 in the group receiving last systemic anticancer treatment in the last 30 days and 6.8 in the group who received last treatment more than 30 days before death (*p* = 0.1).

Finally, a logistic regression analysis was conducted to investigate potential associations between clinical variables and the timing of systemic anticancer therapy administration at the end of life. The model included sex, ECOG performance status status before the last line of systemic therapy, and caregiver presence before the last line of systemic therapy. No statistically significant associations were identified. Male sex: OR = 0.57; *p* = 0.52. Caregiver presence: OR = 1.49; *p* = 0.63. ECOG 2 versus ECOG 1: OR = 1.84; *p* = 0.38.

No statistically significant differences were observed analyzing the characteristics of patients who received systemic therapy between 15 and 30 days before death with those who received it in the last 14 days of life (supplementary Table 1). Once again, respiratory failure was the most common acute event in both groups, occurring in 4 (21%) of patients who received last systemic anticancer treatment between 15 and 30 days before death and in 3 (10%) of those who received it in the last 14 days.

Although there were no significant differences in the types of acute events between the two groups (*p* = 0.09), major bleeding was noted in 5 (25%) of patients receiving last systemic anticancer treatment in the last 14 days, compared to none in the 15–30 days group.

## Discussion

To the authors’ knowledge, this is the first study to analyze a large single-institution cohort of recurrent / metastatic head and neck squamous cell carcinoma patients treated with systemic anticancer therapies at the end of life. While limited by the retrospective design, this study aims to provide valuable insights into the timing and impact of end of life treatments, the use of palliative care, and the occurrence of severe adverse events.

Given that, an initial cohort division (Table 2) was organized to investigate potential differences in OS between those receiving last systemic anticancer treatment in the last 30 days of life and those receiving it earlier. The two groups showed a balanced distribution of clinical characteristics. Subsequently, a second cohort division (Supplementary Table 1) was made in order to further explore end of life systemic anticancer treatments and to compare our work with previous literature data (McPherson et al. 2018).

Survival analysis performed indicates that the group of patients who received last systemic anticancer treatment more than 30 days before death has a better prognosis. Moreover, those who received it within 30 days have also a significant lower PFS1 and lower RR. This finding warrants an extensive interpretation. Since the survival curves reflect the therapeutic pathway from the initiation of the first-line systemic treatment, the observed results may indicate that the cohort of patients with poorer outcomes has more aggressive malignancies, thereby complicating the oncologist's decision on the optimal timing for discontinuing systemic anticancer treatments.

Furthermore, although no statistically significant difference was observed, the group receiving last systemic anticancer treatment within the last 30 days of life demonstrated a minimally average higher N/L ratio compared to the other group, suggesting a potential higher biological aggressiveness and strengthening this theory (Yasumatsu et al. 2019).

Alternatively, if systemic anticancer treatment administered near the end of life directly contributes to earlier mortality, clinical parameters available before initiating the last line of therapy could serve as useful indicators for discontinuation timing. However, in the present analysis, logistic regression did not identify any significant associations between treatment timing and available variables such as ECOG performance status, sex, or caregiver presence.

Further investigation of secondary endpoints is needed to address this issue comprehensively.

Even if no significant difference in fatal acute events was underscored between patients who had undergone systemic therapy within the last 30 and 14 days of their life, major bleeding and respiratory failure were the most frequent fatal acute adverse events registered, as pointed out in (Supplementary Table 1).

In this context, despite the limitations imposed by the small sample size and the lack of statistical significance, it is noteworthy that the group receiving last systemic anticancer treatment in the last 14 days of life experienced 5 episodes of fatal major bleeding, whereas no such events were observed in the group that received it between 30 and 15 days prior to death. To the best of our knowledge, few studies provide detailed information on the precise causes of death in oncology patients receiving end of life systemic anticancer treatments during the last 30 days of life (Haukland et al. 2020). In a population of hospitalized patients with hematologic and solid malignancies, not including head and neck patients, fatal acute events in the last 30 days were observed in 21.7% of cases, compared to 30% in this study, even considering the limitations of the presented data (Haukland et al. 2020). Given that, the high risk of acute events as well as the variety of primary tumor sites, the frequent advanced stage at presentation and the intricate anatomical structures involved, prove that head and neck cancer patients constitute a frail population where the role of the oncologist in deciding end of life systemic anticancer treatments timing is crucial (Johnson et al. 2020). Furthermore, the literature shows that the early use of tertiary palliative care services has a central role in decreasing both suffering and mortality among patients with advanced solid cancer (Temel et al. 2010; Kaasa et al. 2018). Despite the lack of data relating to patients already followed by an outpatient palliative care service, which is the main provider of early palliative care, the analysis shows a higher rate of hospice/home-care referrals compared to what is reported in the literature (78% vs. 47%, respectively). Most requests for admission to hospice or home-care occurred in the last 14 days of life, underscoring the importance of an earlier and shared care pathway management between oncologists and specialized palliative care specialists.

The treatment landscape for HNSCC has evolved significantly in recent years, primarily due to the introduction of immune checkpoint inhibitors such as anti-PD1 agents pembrolizumab and nivolumab (Johnson et al. 2020; Ferris et al. 2016; Cohen et al. 2019). These agents have demonstrated a significant success in clinical trials, leading to their approval for recurrent / metastatic head and neck squamous cell carcinoma. Additionally, immunotherapy has opened new avenues for second-line and subsequent therapies, offering alternatives for patients who have not responded to previous treatments (Johnson et al. 2020; Ferris et al. 2016; Cohen et al. 2019).

Given the favorable toxicity profile, ICIs progressively substituted CT even near the end of life (Golob et al. 2024), with increased use over time in solid cancers, as demonstrated by a recent large retrospective study (Kerekes et al. 2024). Consistently, in the analysis provided nearly half of patients treated during the last 30 days of life received immunotherapy as systemic anticancer treatment, confirming this evolving paradigm (Canavan et al. 2022).

In this scenario, the incidence of end of life systemic anticancer treatments in our cohort was numerically lower than figures reported in historical data across all cancer types, both for administration within the last 30 days (25% vs. 39%) and within the last 14 days of life (13% vs. 17%) (Canavan et al. 2022; McPherson et al. 2018; Parikh et al. 2019; Riaz et al. 2020). However, due to the number of excluded patients and the absence of direct statistical comparison, this observation should be interpreted with caution.

This study presents several limitations. First, it is a retrospective, monocentric analysis based on a relatively small sample size, limiting the representativeness of the findings. Second, a substantial proportion of patients were excluded due to missing data, potentially affecting internal validity. Third, some relevant clinical variables—such as tumor invasion of critical anatomical structures (e.g. carotid artery)—were not available and thus could not be included in the analysis. Additionally, the logistic regression model was based on a limited number of pre last line of treatment clinical variables, which may have reduced its predictive power.

Nevertheless, this study provides a comprehensive analysis of systemic anticancer treatments utilization in recurrent / metastatic head and neck squamous cell carcinoma cancer, fatal acute events, and residential palliative care request rates, thus addressing a literature gap. The comparison between fatal acute events when last systemic anticancer treatment was administered between the last 30 and 15 days of life and within the last 14 days raises the debated issue of the role of end of life systemic anticancer treatments in facilitating episodes of major bleeding. Patients who received systemic anticancer treatment in the last 30 days of life may be affected from more aggressive subtypes of recurrent / metastatic head and neck squamous cell carcinoma cancer, making the finding of exactly treatment stopping time challenging. These data might suggest the efficacy of a knowledgeable patient selection, a high level of clinical expertise in managing patients with head and neck cancer, and a multidisciplinary discussion of the case, in particular with the palliative care units.

## Conclusions

This monocentric retrospective analysis of recurrent / metastatic head and neck squamous cell carcinoma patients found that patients whose last systemic anticancer treatment occurred more than 30 days before death experienced longer survival; this association likely reflects underlying differences in disease aggressiveness. These findings highlight the complexity of clinical decision-making regarding the timing of end-of-life treatments. Compared to previously published data, the observed incidence of end-of-life systemic therapies was lower, and palliative care referrals were relatively frequent, potentially reflecting a multidisciplinary approach to care in a tertiary setting. Respiratory failure and major bleeding were the most common fatal acute events, underlining the clinical challenges in this frail population. While fatal major bleeding episodes appeared more frequent when treatment was administered within 14 days of death, this observation did not reach statistical significance and warrants further investigation. In a rapidly evolving oncological landscape, these data may contribute to informing future clinical decisions regarding systemic treatment near the end of life.

## Supplementary Information

Below is the link to the electronic supplementary material.

Supplementary Material 1

## Data Availability

No datasets were generated or analysed during the current study.
