# Supplementary material for: Systemic anticancer therapy during end of life in head and neck squamous cell carcinoma patients. A retrospective single center study
Source: J Cancer Res Clin Oncol. 2025 Aug 29;151(8):240. doi: 10.1007/s00432-025-06297-5 (PMC12397045; doi:10.1007/s00432-025-06297-5)
Supplement: Supplementary file 1 — Supplementary Material 1 [file 432_2025_6297_MOESM1_ESM.docx]

| **Characteristics** | **Last systemic anticancer treatment between 15 and 30 days, N = 19** | **Last systemic anticancer treatment during last 14 days, N = 20** | **p-value^2^** |
| --- | --- | --- | --- |
| Gender |  |  | 0.4 |
| Female | 6 (32%) | 9 (45%) |  |
| Male | 13 (68%) | 11 (55%) |  |
| Baseline ECOG |  |  | 0.4 |
| 1 | 13 (68%) | 17 (85%) |  |
| 2 | 6 (32%) | 3 (15%) |  |
| Acute events |  |  | >0.9 |
| yes | 8 (42%) | 9 (45%) |  |
| no | 4 (21%) | 5 (25%) |  |
| Data not available (NA) | 7 (37%) | 6 (30%) |  |
| Type of Acute event |  |  | 0.09 |
| Respiratory failure | 4 (21%) | 2 (10%) |  |
| Major bleeding | 0 (0%) | 5 (25%) |  |
| Sepsis | 3 (16%) | 1 (5%) |  |
| Stroke | 1 (5%) | 0 (0%) |  |
| Pulmonary thromboembolism | 0 (0%) | 1 (5%) |  |
| NA | 11 (58%) | 11 (55%) |  |
| Type of last therapy |  |  | 0.4 |
| Chemotherapy | 8 (42%) | 10 (50%) |  |
| Immunotherapy | 8 (42%) | 10 (50%) |  |
| Target therapy | 1 (5%) | 0 (0%) |  |
| NA | 2 (11%) | 0 (0%) |  |
| Presence of a caregiver |  |  | 0.5 |
| Yes | 16 (84%) | 14 (70%) |  |
| No | 3 (16%) | 6 (30%) |  |
| 1n (%); Median (IQR) | | | |
| 2Fisher's exact test; Welch Two Sample t-test; Pearson's Chi-squared test | | | |

Supplementary table 1: Patients characteristics in two different timing of end of life systemic anticancer treatment (30 - 15 vs 14 – death)
